# Asserting Accountability to Address Diversity: Report Card as a System of Measurement

**DOI:** 10.1089/heq.2021.0169

**Published:** 2023-02-20

**Authors:** Corrinne Fahl, Dominique Alexis, Eve J. Higginbotham, Chang Xu, Jaya Aysola

**Affiliations:** ^1^ Office of Inclusion, Diversity, and Equity, Perelman School of Medicine, University of Pennsylvania, Philadelphia, Pennsylvania, USA.; ^2^ Office of Inclusion, Diversity, and Equity, Department of Ophthalmology, Perelman School of Medicine, University of Pennsylvania, Philadelphia, Pennsylvania, USA.; ^3^ Perelman School of Medicine, University of Pennsylvania, Philadelphia, Pennsylvania, USA.; ^4^ Division of General Internal Medicine and Office of Inclusion, Diversity, and Equity, Perelman School of Medicine, University of Pennsylvania, Philadelphia, Pennsylvania, USA.

**Keywords:** inclusion, diversity, metrics, diversity spending

## Abstract

**Problem:**

To the best of our knowledge, there are no standard accountability measures for diversity efforts at the departmental level. Therefore, the purpose of this study is to evaluate a multiprong report card as a structure for evaluation, tracking, and reporting as well as to examine any relationships between expenditures and outcomes.

**Approach:**

We instituted an intervention that offered leadership a report card of metrics related to diversity efforts. Included are diversity expenditures, benchmark demographic and departmental data, applications to support faculty salaries, participation in clerkship programs focused on attracting diverse candidates, and requests for candidate lists. The purpose of this analysis is to demonstrate the impact of the intervention.

**Outcomes:**

A significant relationship was found between faculty funding applications and under-represented minority (URM) representation in a department (0.19; confidence interval [95% CI] 0.17–0.21; *p*<0.001). An association was also found between total expenditures and URM representation in a department (0.002; 95% CI 0.002–0.003; *p*<0.001). Other outcomes include the following: (1) women, URM, and minority faculty have all increased in representation since tracking began; (2) diversity expenditures and faculty opportunity fund and presidential professorship applications have increased over time; and (3) a steady decline in departments with zero URM representation after the tracking of diversity expenditures in both clinical and basic science departments.

**Next Steps:**

Our findings suggest that standardized metrics for inclusion and diversity initiatives promote accountability and buy-in from executive leadership. Departmental detail enables tracking of progress longitudinally. Future work will continue to evaluate the downstream effects of diversity expenditures.

## Introduction

Accountability is a means by which institutional culture can change.^1^ Creating and promoting a culture of institutional accountability begin with the leadership level.^1,2^ Applying accountability to diversity and inclusion efforts can guide academic medical institutions toward a more positive culture. There exist many diversity initiatives, but a thorough literature search has not shown a standardized method of evaluating these efforts. As a component of institutional stewardship, we must be able to analyze where a program has been, where it is headed in the future, the type of impact, and ultimately help determine if goals are being met.^1^

This level of accountability specifically around inclusion and diversity is largely absent from academic medicine literature. Academic institutions and hospital systems have outlined various metrics to increase inclusion and diversity.^2^ These include recruitment, retention,^3^ exit interviews, advancement, and workforce representation. One area of focus that deserves more attention is metrics on expenditures, and other concrete indicators of departmental investment related to inclusion and diversity efforts. Research around these programs^4,5^ has not focused on analyzing relationships between initiatives and outcomes, with the exception of bias training.

While there are some analyses making the business case for diversity and discussing the financial benefits to corporations and institutions of these efforts,^6,7^ the connection to spending is not directly addressed.^5^ To the best of our knowledge, little is known about the intersection of department expenditures and diversity. Further understanding of how expenditures play a role in departments provides a unique opportunity. Therefore, the purpose of this study was to evaluate the effectiveness of these metrics in assessing the relationship between diversity expenditures and increased faculty diversity.

## Approach

Our intervention aims to systemize and track departmental-level efforts in diversity to ensure accountability across the institution. Previous literature that has examined evaluating inclusion and diversity programs in business organizations indicates the importance of metrics to improve those programs and to facilitate buy-in at the executive level.^6,7^ To accomplish this goal, we developed this report card. The tracking of diversity-related efforts is a component of the annual department chair review process.

Before the creation of the report card, there was not a fine-grained longitudinal examination of these efforts included in the annual department chair reviews. The report card does not distinguish between programs that existed previously and those that are newly created. It is provided to the Dean of the Perelman School of Medicine before the annual department chair reviews. This includes comparisons of all clinical departments and all basic science departments. This information is shared with the department chairs rather than directly with any group of under-represented minority (URM) faculty, and the expenditures report is filled out by the department chair in concert with their business office. The primary focus of these report cards is to have longitudinal data and accountability for the department leadership.

### Tracking and reporting diversity expenditures

The University of Pennsylvania instituted reporting of expenditures related to inclusion and diversity in fiscal year (FY) 2011 in conjunction with Penn's Diversity Action Plan and Budget Initiative. The first Institutional Diversity Expenditures Budget to include school-level expenditures was developed in FY 2013. The same year, the report was revised to contain department-level data. The original purpose of the diversity expenditures report was to track the school-level efforts in different diversity activities across time. Department-level data were added shortly after. There were no predetermined outcomes being tracked.

The Penn Compact 2020, launched in 2013, focused on the following principals: Increase Access, Integrate Knowledge, and Engage Locally, Nationally, and Globally, at the same time that school-level diversity expenditures began to be reported. The reporting template was redesigned in FY 2014, and the production of the report became a joint effort between Faculty Affairs and Professional Development (FAPD) and the Office of Inclusion, Diversity, and Equity (OIDE) in the Perelman School of Medicine. Beginning in FY 2017, OIDE developed a system of metrics including the diversity expenditures to be used in annual department reviews.

**FIG. 1.**
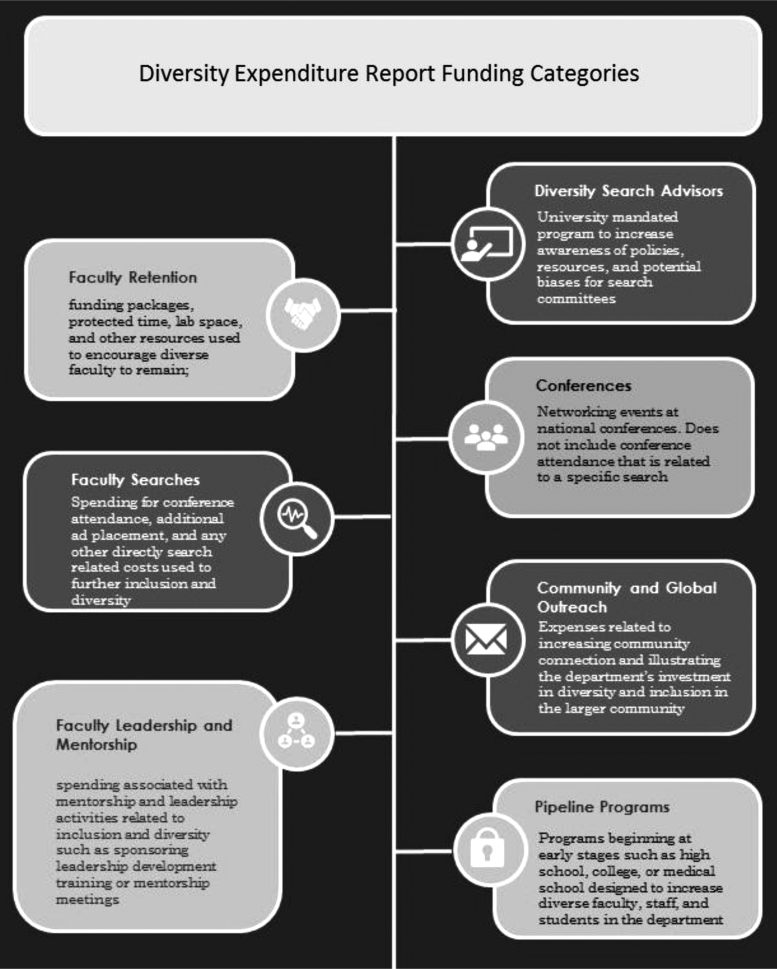
Diversity expenditures report funding categories.

Departmental-level data included in the diversity expenditures report are broken down into the following categories: (1) faculty retention, (2) diversity search advisors, (3) faculty searches, (4) faculty leadership and mentorship, (5) conferences, (6) community and global outreach, (7) and pipeline programs (Fig. 1). These metrics allow leadership to comparatively evaluate departments and reveal opportunities for improvements. As a component of annual department reviews, the metrics created incentives for department chairs to make measurable efforts to increase its departmental commitment to inclusion and diversity recruitment and retention. In addition to the financial measures of diversity efforts, the following areas are included in the annual reporting:

### Benchmark faculty demographic data

Faculty demographics for the department and in comparison, to the rest of the medical school, are provided to the chair and the Dean as part of the report card. These data also include the average demographics of the specialty by rank from the American Association of Medical College's (AAMC) U.S. Medical School Faculty Report, most recently published with 2019 data,^8^ and diversity of the residency program. These data allow each department to evaluate itself by demographics on a national level. URM includes Black, Hispanic/Latinx, Native American, and Pacific Islander. Minority includes these categories and additionally includes Asian faculty.

### Applications to support faculty salaries

In addition to diversity expenditures, the evaluation of departments includes applications for two university funds for diverse candidates: (1) faculty opportunity fund (FOF) and (2) presidential professorships (PPs) (Fig. 1). The FOF and the PPs are funded centrally by the university, and applications are competitive among all the schools. The FOF was launched in 2006, and the Presidential Professorship Program was created in 2011 as part of Penn's Action Plan for Faculty Diversity and Excellence. Both programs support faculty salary for 5 years.

Both sources of funding are tools to support department recruitment of diverse candidates, by defraying a portion of the salary cost. As a result, we used the number of FOF and PP applications as the metric. The submission of applications indicates a level of commitment and engagement on the part of the department; therefore, we used this as the metric rather than the number of approved applications. OIDE and FAPD actively reach out and provide support to departments regarding applications for these two university-funded programs. This information has been included in the report card since its inception.

### Faculty candidate database requests

Departments have the option to request lists of diverse faculty candidates from OIDE, a program that is itself novel among academic medical centers. Departments supply OIDE with the track, rank, specialty, and any other critical information about the position, and a list of candidates is supplied from peer institutions and institutions that graduate high numbers of URM physicians.

OIDE examines public pages of faculty at peer institutions and institutions, which produce higher numbers of URM doctors, and creates a list based on departmental criteria. This information is not provided to OIDE by the external institution. This service has been available to departments since FY17. The number of requests made is included in the annual reporting. Requests are made by rank, track, and specialty. The requests have been part of the report card since FY18.

### Participation in Clerkship Programs

The Perelman School of Medicine URM Visiting Clerkship Program is a program developed by the Alliance for Minority Physicians partnered with the University of Pennsylvania Health System and The Children's Hospital of Pennsylvania (CHOP) in FY11. This program offers fourth-year medical students, nationally, the opportunity to explore rotations at CHOP and many of the Penn-affiliated hospitals. Students have access to mentorship with residents and faculty, funding for their clerkship, and the chance to meet with program directors. This program is a component of the overall evaluation, and participation in this program has been noted as a metric in the report card since FY17.

### External department review

When preparing documentation for external review of the department, the average demographics of the specialty by rank, demographics of the department by rank, and diversity of the residency program are provided to the committees, in addition to the expenditure and funding application data. External department reviews take place every 5–7 years, by a committee selected by the Dean.

The schools are periodically reviewed by a committee appointed by the President and Provost. This meets the Middle States requirements for continuing accreditation, which states, “The institution's planning processes, resources, and structures are aligned with each other and are sufficient to fulfill its mission and goals, to continuously assess and improve its programs and services, and to respond effectively to opportunities and challenges.”^9^

### Statistical analysis

Our analysis had three main objectives: first, we determined distributions of each characteristic of our report card and outcomes of interest. Second, we compared trends for these measures across years for all available data. Third, we evaluated associations between our report card components and the number of URM faculty. We tabulated and compared the distributions (mean [standard deviation], median [interquartile range]) of each element for basic science and clinical departments, using the nonparametric Wilcoxon rank-sum tests. In our adjusted analyses, our dependent variable was URM faculty count.

Our key independent variables included the following: (1) diversity expenditures; (2) FOFs and/or PPs (FOF/PP); (3) department type (Clinical vs. Basic Science); and time trend (FY16 through FY19). We estimated the relationship between our independent and dependent variables using negative binomial regression models to account for overdispersion in the URM faculty count outcome and generalized estimating equations with an exchangeable correlation structure to account for covariate clustering by year. Two-tailed *p*-values and confidence intervals (95% CIs) are reported for all statistical tests, with *p*<0.05 considered statistically significant.

## Outcomes

**FIG. 2.**
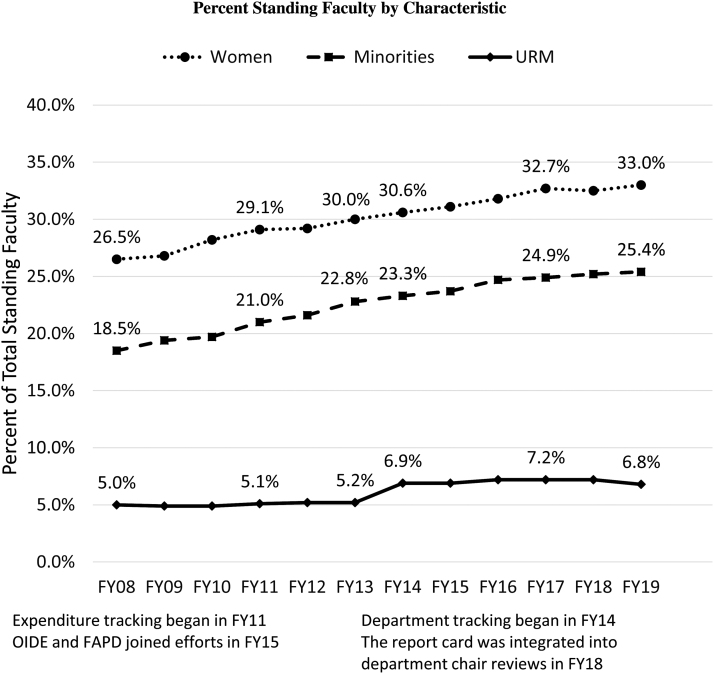
Percentage standing faculty by characteristic percentage. URM includes Black, Hispanic/Latinx, Native American, and Pacific Islander. Minority includes these categories and additionally includes Asian faculty. FAPD, Faculty Affairs and Professional Development; FY, fiscal year; OIDE, Office of Inclusion, Diversity, and Equity; URM, under-represented minority.

Although the basic science departments are smaller than clinical departments, the representation of diverse candidates was noted to be not statistically significant between these two groups (Table 1). However, representation of women, URM, and minorities who are standing faculty has all increased since expenditure tracking began in FY11 (Fig. 2 and Table 2). Standing faculty includes tenure track faculty and clinician educator track faculty. These two groups engage in research, teaching, and clinical activity.

**Table 1.** Basic and Clinical Department Characteristic Comparisons by Fiscal Year

	Basic science (***N***=10)	Clinical (***N***=18)	***p***
FY16			
Total dept. size			
*N*	10	18	0.0004
Mean (SD)	22.2000 (10.7683)	114.3889 (121.1922)	
Median	19.5	55.0	
Q1, Q3	15.0, 25.0	41.0, 149.0	
Range	(13.0–49.0)	(20.0–464.0)	
Total expenditures			
*N*	7	18	0.7165
Mean (SD)	$344903.143 ($254748.957)	$1148841.56 ($1499021.39)	
Median	$233145.0	$270012.5	
Q1, Q3	$106430.0, $670259.0	$126630.0, $2099295.0	
Range	($90000.0–$689887.0)	($34400.0–$4552471.0)	
URM %			
*N*	10	18	0.1189
Mean (SD)	0.0541 (0.0493)	0.0811 (0.0395)	
Median	0.1	0.1	
Q1, Q3	0.0, 0.1	0.1, 0.1	
Range	(0.0–0.2)	(0.0–0.2)	
Women %			
*N*	10	18	0.7552
Mean (SD)	0.3324 (0.1102)	0.3506 (0.1446)	
Median	0.3	0.4	
Q1, Q3	0.3, 0.4	0.2, 0.4	
Range	(0.2–0.6)	(0.1–0.7)	
FOF and PP			
*N*	10	18	0.0321
Mean (SD)	0.1000 (0.3162)	0.7222 (0.8264)	
Median	0.0	0.5	
Q1, Q3	0.0, 0.0	0.0, 1.0	
Range	(0.0–1.0)	(0.0–2.0)	
FY 17			
Total dept. size			
*N*	10	18	0.0009
Mean (SD)	23.5000 (11.2867)	119.4444 (128.3393)	
Median	22.0	54.0	
Q1, Q3	16.0, 27.0	45.0, 156.0	
Range	(13.0–51.0)	(18.0–482.0)	
Total expenditures			
*N*	10	18	0.7372
Mean (SD)	$612041.310 ($650397.592)	$820549.150 ($1068670.37)	
Median	$353672.0	$208464.5	
Q1, Q3	$205804.1, $736154.0	$60807.0, $1347942.4	
Range	($0.0–-$1823032.0)	($7147.9–$3482599.0)	
URM %			
*N*	10	18	0.1566
Mean (SD)	0.0549 (0.0442)	0.0852 (0.0493)	
Median	0.1	0.1	
Q1, Q3	0.0, 0.1	0.1, 0.1	
Range	(0.0–0.1)	(0.0–0.2)	
Women %			
*N*	10	18	0.5979
Range	(0.2–0.5)	(0.1–0.6)	
FOF+PP			
*N*	10	18	0.2698
Mean (SD)	0.3000 (0.6749)	0.7222 (1.0178)	
Median	0.0	0.0	
Q1, Q3	0.0, 0.0	0.0, 2.0	
Range	(0.0–2.0)	(0.0–3.0)	
FY18			
Total dept. size			
*N*	10	18	0.0008
Mean (SD)	24.4000 (10.7724)	125.8889 (135.6292)	
Median	22.0	57.5	
Q1, Q3	19.0, 27.0	47.0, 162.0	
Range	(13.0–52.0)	(18.0–507.0)	
Total expenditures			
*N*	10	18	0.6661
Mean (SD)	$511214.600 ($562099.169)	$1118561.79 ($1595480.70)	
Median	$241383.0	$346911.5	
Q1, Q3	$210303.0, $566837.0	$121987.0, $1670079.3	
Range	($14253.0–$1720000.0)	($18400.0–$5982565.0)	
URM %			
*N*	10	18	0.1952
Mean (SD)	0.0626 (0.0476)	0.0894 (0.0485)	
Median	0.1	0.1	
Q1, Q3	0.0, 0.1	0.1, 0.1	
Range	(0.0–0.2)	(0.0–0.2)	
Women %			
*N*	10	18	0.5489
Mean (SD)	0.3445 (0.1065)	0.3719 (0.1525)	
Median	0.3	0.4	
Q1, Q3	0.3, 0.4	0.2, 0.5	
Range	(0.2–0.5)	(0.1–0.7)	
FOF+PP			
*N*	10	18	0.6624
Mean (SD)	0.3000 (0.4830)	0.6667 (1.2834)	
Median	0.0	0.0	
Q1, Q3	0.0, 1.0	0.0, 1.0	
Range	(0.0–1.0)	(0.0–5.0)	
FY 19			
Total dept. size			
*N*	10	18	0.0007
Mean (SD)	25.2000 (11.0735)	132.8333 (144.5314)	
Median	22.0	61.0	
Q1, Q3	20.0, 29.0	49.0, 168.0	
Range	(13.0–53.0)	(20.0–546.0)	
URM %			
*N*	10	18	0.0390
Mean (SD)	0.0521 (0.0436)	0.0860 (0.0452)	

dept., department; FOF, faculty opportunity fund; PP, presidential professorship; SD, standard deviation; URM, under-represented minority.

**Table 2.** Change in Faculty Over time

Date/year	Full-time faculty total	URM full-time faculty	Standing faculty total	URM standing faculty	Academic faculty	URM academic faculty
June 30, 2013	1982	117	1362	79	455	34
June 30, 2021	2883	206	1548	92	1158	86
Total change	901	89	186	13	703	52
% change	45.5	76	13.6	16.5	154	153

Full-time faculty include all assistant, associate, and full professors with the exception of adjunct. Standing faculty include tenure track or clinician educator. Academic faculty include academic clinicians; URM includes Black, Hispanic/Latinx, Native American, and Pacific Islander. Minority includes those included in URM as well as Asian.

URM, under-represented minority.

**FIG. 3.**
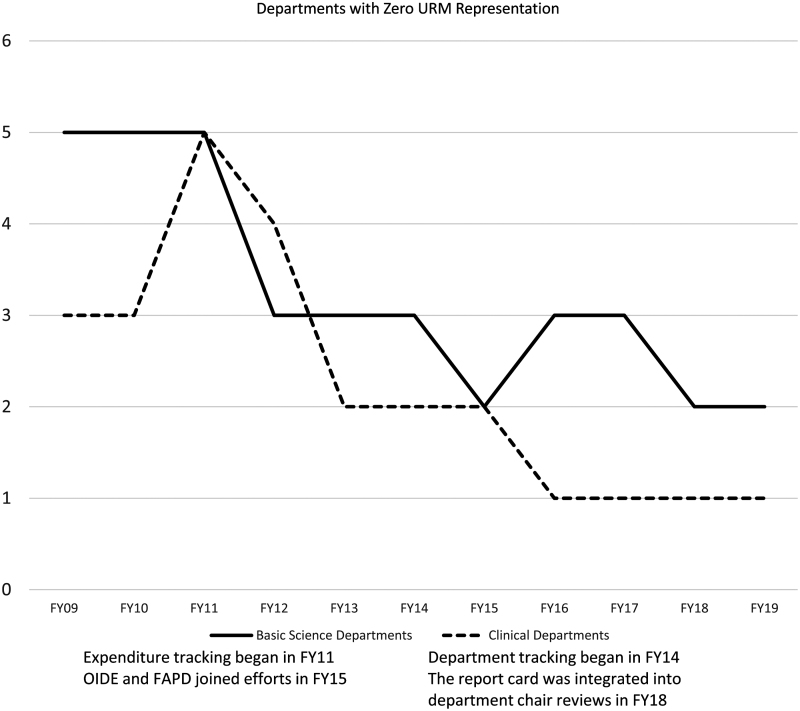
Departments with zero URM representation. URM includes Black, Hispanic/Latinx, Native American, and Pacific Islander. Minority includes these categories and additionally includes Asian faculty.

The number of departments with no URM faculty has decreased since tracking began in FY11 (Fig. 3), with additional improvements in the clinical departments shown after departmental-level tracking began, and after the tracking became a collaboration between OIDE and FAPD. After adjusting for time trend (FY16 through FY19) and department type (Clinical vs. Basic Science), we found a significant relationship between applications for FOFs and/or PPs and the number of URMs in a department (0.19; 95% CI 0.17–0.21; *p*<0.001).

In addition, there was a significant association between total expenditures and the number of URMs in a department (0.002; 95% CI 0.002–0.003; *p*<0.001) after adjusting for year (FY17 and FY18) and department type. Clinical departments have shown a sustained interest in resources such as the faculty candidate database and FOF and PP, while the basic science departments noticeably increased their requests in FY19 (Table 1).

Despite basic science departments' low engagement with the faculty candidate database, they have shown an increase in applications (Table 1). It should also be noted that a retrospective study of changes in medical school faculty demographics by Xierali et al^10^ found that clinical departments observed significantly more change in URM faculty compared with basic science departments.

Inclusion and diversity initiatives are becoming more common in medical schools across the country.^4^ However, the primary metric used to evaluate them in academic centers, if any, is student and faculty demographics,^1^ which is not tied directly to accountability for department leadership. Demographic change is an important objective, but it does not measure all efforts equally, and it may discourage programmatic initiatives that are not demonstrating an immediate yield.

It can also be challenging to tie a specific initiative directly to increased diversity, as demographics are a lagging indicator. Using a method of evaluation that accounts for effort means that schools can fairly assess a department's commitment to change. The data are also supplied to the external committees during the regular process of department reviews. This high-level review is not in place in any other academic medical institution, to the best of our knowledge.

### Strengths and limitations

We acknowledge some limitations to our study. Our findings may not be nationally generalizable, given that these are data obtained from one institution. However, the data included all departments in the medical school, over a period of 4 years, which allows us to see larger trends without overemphasis on any given specialty. Another limitation is that only 4 years of data were available for analysis, which is challenging given that results related to pipeline and outreach efforts can take years to manifest. Because pipeline programs and outreach can start quite early, effects may not be truly visible for quite some time.

Rather than attempting to measure the effectiveness of these programs, we examine the commitment of the department to these programs. However, using the same format for reporting revealed consistent results and a strong framework to use for further evaluation. Finally, to our knowledge, this analysis is the first of its kind at an academic center, and provides a holistic evaluation of inclusion and diversity efforts.

The report card does not include funding used for patient care, but the expenditures report does include pipeline programs and outreach, as well as an “other” category left to the department's discretion, which may include educational spending.

Metrics were developed to track expenditures related to inclusion and diversity efforts over time. These metrics have been successfully used in the review of departments, both internally and externally. Using the same metrics across all departments allows us to identify leaders and offer assistance where it is most needed. Developing metrics that expanded upon the original expenditure tracking program creates a broader view of the efforts made by departments. The addition of these data in the annual chair evaluations has created an opportunity for an ongoing conversation with departmental leadership about the importance of all their inclusion and diversity programs, and the recruitment and retention of diverse faculty.

The school has a way to hold the departments accountable, which is transparent to the department chairs. It is important to include metrics that are tied to efforts, rather than only to outcomes. By doing so, we recognize the work being done in departments that may not yet have reached their demographic goals but are investing in long-term diversity.

## Next Steps

Our study reveals promising results utilizing metrics to assess the effectiveness of diversity and inclusion efforts, from a financial standpoint. With a standardized approach, we can better track our progress and highlight areas that need improvement. The strategic plan includes continuing to collect these data every year, and analyze trends in the relationship between expenditures and increased diversity as well as continuing to evaluate the categories of reporting, and to ensure that departments are using the classifications consistently so that comparison is possible.

In addition, pipeline programs will have the opportunity to bear fruit, and use this blueprint to examine costs and outcomes related to their program development. Fundamentally, we have found that measuring actions rather than outcomes indicates a connection between those efforts and increased URM faculty. We have the opportunity to encourage further efforts, to acknowledge the current efforts in departments, and to demonstrate the importance of institutional buy-in and support.

## Abbreviations Used

AAMC: American Association of Medical College
CHOP: Children's Hospital of Pennsylvania
CI: confidence interval
FAPD: Faculty Affairs and Professional Development
FOF: faculty opportunity fund
FY: fiscal year
OIDE: Office of Inclusion, Diversity, and Equity
PP: presidential professorship
URM: under-represented minority
